# Probing Ultrafast Excitonic Coherences and Charge-Generation Pathways in Quantum-Dot Photocells via Photocurrent-Detected Two-Dimensional Electronic Spectroscopy

**DOI:** 10.1021/acsnano.6c04829

**Published:** 2026-06-22

**Authors:** Hadar Manis Levy, Matilde Doardo, James R. Hamilton, Carlo Nazareno Dibenedetto, Thibault Degousée, Jan A. Mol, Marinella Striccoli, Francoise Remacle, Elisabetta Collini

**Affiliations:** † Dipartimento di Scienze Chimiche, 9308 Università degli Studi di Padova, Padova 35131, Italy; ‡ Theoretical Physical Chemistry,Research Unit Molecular Systems, 26658 University of Liege, Liege B4000, Belgium; § CNR-IPCF SS Bari, c/o Chemistry Department, 201810 University of Bari Aldo Moro, Via Orabona 4, Bari 70126, Italy; ∥ School of Physical and Chemical Sciences, Queen Mary University, London E1 4NS, U.K.; ⊥ The Fritz Haber Center for Molecular Dynamics and Institute of Chemistry, The Hebrew University of Jerusalem, Jerusalem 91904, Israel

**Keywords:** photocurrent-detected 2DES, quantum-dot photocells, excitonic coherence, charge separation, interdot coupling, ultrafast spectroscopy

## Abstract

Understanding how coherent excitonic states influence charge generation is essential for optimizing quantum-dot (QD) optoelectronic devices. Here, we apply photocurrent-detected two-dimensional electronic spectroscopy (PC-2DES) to a functioning CdSe QD photocell to track excitonic interactions and charge-separation dynamics under operational conditions. Selective excitation of the |1S⟩ manifold reveals strong contributions from red-shifted, delocalized excitons that dominate the photocurrent but contribute only weakly in optically detected 2DES. Global analysis uncovers three dynamical components, including a sub-100 fs process associated with photocurrent growth at specific spectral coordinates, consistent with rapid population transfer through delocalized states and possible trion formation. Unlike optical detection, PC-2DES suppresses longitudinal optical phonon signatures, enabling clear observation of higher-frequency beatings attributed to interdot electronic coherences. These results demonstrate the utility of PC-2DES for probing coherent charge-generation pathways in QD solids and for guiding the design of coherence-enabled optoelectronic devices.

## Graphical abstract

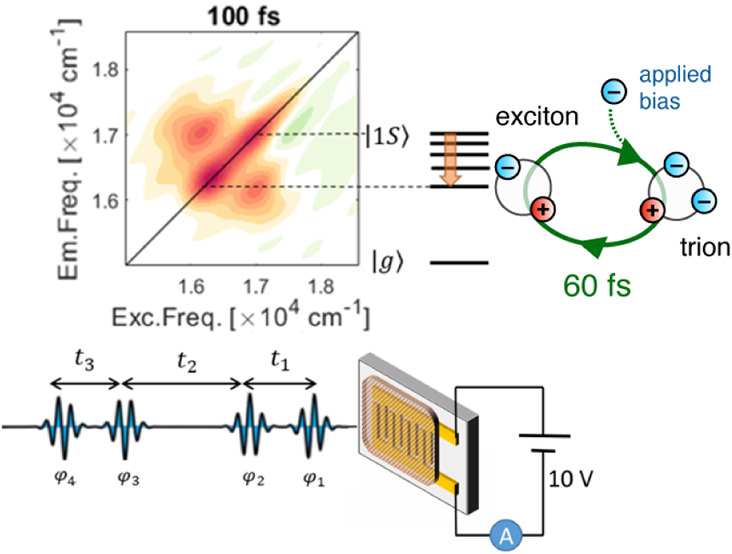

## 1 Introduction

Quantum technologies are rapidly emerging as a transformative field, offering capabilities that surpass the limits of classical systems in areas such as computation, sensing, and secure communication.^1,2^ Central to these advancements is the concept of quantum coherence, which underpins the controlled manipulation of quantum states necessary for reliable device operation. Semiconductor quantum dots (QDs), owing to their discrete energy levels and tunable properties, have become a versatile platform for studying coherent quantum phenomena and for developing solid-state quantum devices.^3^ The field is now transitioning from fundamental investigations of quantum behavior toward the realization of scalable, application-oriented technologies.^4–7^ This shift, however, brings to the forefront critical challenges, including size-dependent effects that influence coherence times and functional integration. Addressing these issues is essential for the development of robust electro-optic devices that exploit quantum effects for enhanced performance in real-world applications. In this context, the use of cutting-edge characterization techniques, such as two-dimensional electron spectroscopy (2DES), is crucial for probing ultrafast dynamics and coherence properties with high spectral and temporal resolution, thereby guiding the rational design of next-generation quantum systems.^8,9^

To this end, our group has employed 2DES to explore coherent phenomena in QDs. Initial studies focused on QDs dispersed in solution,^4^ where we unambiguously demonstrated the time evolution of specific coherent superpositions of fine-structure levels localized within individual QDs, referred to as *intradot* electronic coherences. Building on these findings, we extended our investigations to QDs in solid state assemblies.^5,10^ In this regime, we observed the time evolution of coherent superpositions of electronic states delocalized across multiple QDs, known as *interdot* electronic coherences, again under ambient conditions. Transitioning from isolated QDs in solution to condensed-phase films—where QDs form dimeric or aggregated structures—resulted in a redistribution of oscillator strength and a corresponding reshaping of the band structure, particularly on the low-energy side of the |1S⟩ exciton band. This result provides insight into how coherence and excitonic interactions evolve in realistic, device-relevant environments.^7,11,12^

While conventional coherently detected 2DES (C-2DES) in a noncollinear geometry has provided deep insights into ultrafast coherence dynamics by isolating coherent optical signals via spatial phase matching (Figure 1a), it does not directly reflect the operational behavior of electro-optic devices, which depend on complex processes such as charge separation and carrier transport. To bridge this gap, a complementary class of techniques, collectively known as *action*-based or *population*-based 2DES, has emerged.^13–16^ These approaches rely on detecting incoherent observables arising from excited-state populations, rather than on the coherent optical response. By introducing a fourth pulse that converts the third-order polarization into a fourth-order population, these methods enable the detection of signals associated with physical outcomes such as photocurrent (PC),^17,18^ photoelectrons,^19^ photoions,^20^ or fluorescence,^13^ depending on the system and target observable. Among them, photocurrent-detected 2DES (PC-2DES) stands out for its ability to directly monitor charge carriers extracted from a functioning device (Figure 1b), thereby linking ultrafast quantum coherence to practical device performance. Since the PC signal reflects real processes of charge separation, transport, and collection, PC-2DES provides direct access to how coherent phenomena influence charge dynamics in operational optoelectronic systems.^18,21–24^ Despite the promise of PC-2DES for linking fundamental quantum dynamics to device-level functionality, experimental implementations of this technique are still relatively scarce.

**Figure 1:**
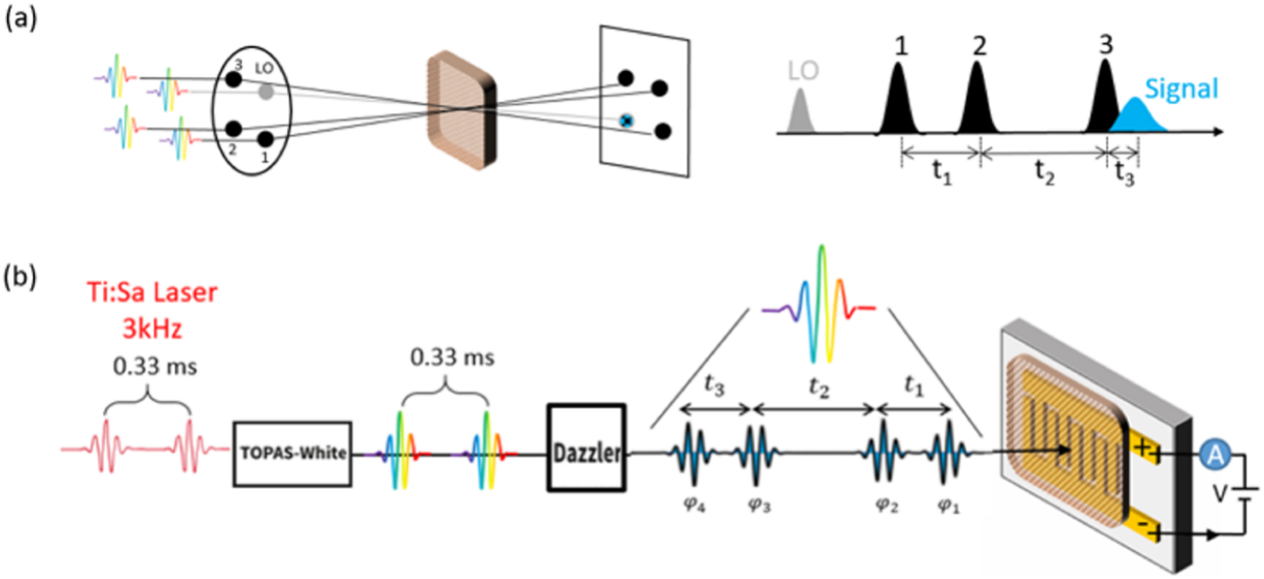
Comparison between coherently detected 2DES (C-2DES) and photocurrent-detected 2DES (PC-2DES). (a) C-2DES employs a noncollinear boxCARS geometry to isolate phase-matched third-order signals originating from the macroscopic polarization, enabling the characterization of ultrafast coherence and population dynamics. (b) PC-2DES utilizes a collinear sequence of four phase-modulated pulses to generate and detect a fourth-order population response via the photocurrent output of an operational device. This action-based detection scheme directly links coherent excitonic and charge dynamics to measurable device performance parameters, such as carrier extraction and transport.

In this study, we investigate charge transport dynamics in a CdSe QD photocell using the PC-2DES technique. PC-2DES has previously been applied to QD photocells by Karki et al.,^24^ who demonstrated its capability to probe subpicosecond dynamics associated with multiple exciton generation and excitation-induced spectral shifts in PbS QDs. While these earlier studies primarily focused on many-body effects under high-energy excitation conditions, here we employ PC-2DES to investigate ultrafast excitonic coherences and their direct connection to charge-generation pathways under near-band-edge excitation. This approach enables us to identify the specific excitonic states contributing to PC generation.

The photocell studied here is based on solid-state thin films of QDs comparable to those used in our earlier work with C-2DES,^5^ enabling qualitative comparison between the coherence pathways associated with purely optical responses and those arising from electrically detected charge transport processes in PC-2DES. This comparison reveals distinct transition pathways that emerge due to electrical charge transfer, offering new insights beyond those accessible with purely optical probes.

## 2 Results and Discussion

### 2.1 Device Design and Linear Characterization

The PC-2DES experimental setup employed a sequence of four collinearly aligned femtosecond pulses, precisely delayed and phase-modulated using an acousto-optic pulse shaper, to excite the photocell device and induce a measurable photocurrent as in ref 22 (Figure 1b). The excitation pulses were spectrally tuned to cover the lowest-energy electronic transitions (Figure 2), consistent with previous studies,^5^ and had a temporal duration of approximately 10 fs. The PC signal, measured at ambient conditions and under an applied bias of 10 V,^25,26^ was monitored as a function of the time delays between the first and second pair of pulses (*t*_1_ and *t*_3_), which set the excitation and detection frequencies (ω_1_ and ω_3_), respectively, whereas the population time *t*_2_ fixes the time interval between excitation and detection pairs. A phase-modulation protocol was employed in which the phases of the individual pulses were modulated in time to isolate the different components of the spectroscopic response, enabling the retrieval of both the linear and the fourth-order rephasing and nonrephasing signals within a single measurement (see Methods).^23^ A known artifact arising from the nonlinearity of the Dazzler power^27^ was corrected through postprocessing of the data by rescaling the measured signal using a correction factor obtained from independent photodiode measurements of the pulse sequence acquired in both the linear and nonlinear operating regimes of the pulse shaper, as detailed in the Supporting Information, Figure S1. To minimize multiparticle interactions, all measurements were conducted at sufficiently low excitation intensities (see Supporting Information, Figure S2).

**Figure 2:**
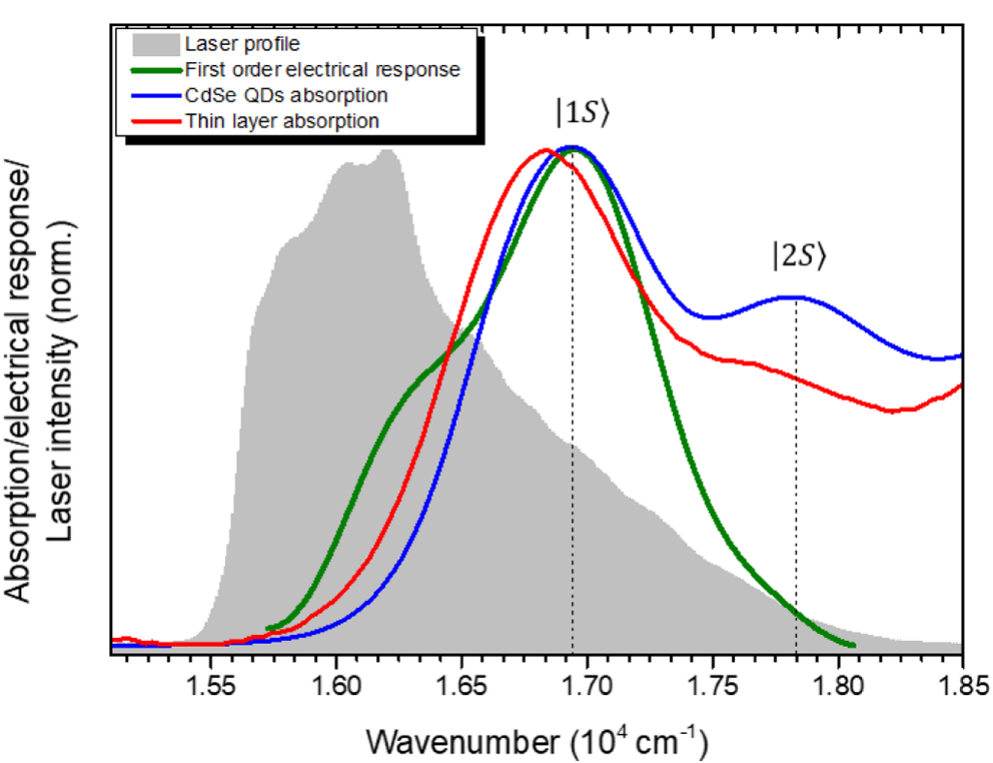
Optical characterization of CdSe quantum dots (QDs). Linear absorption spectra of the CdSe QD sample in solution (blue) and as a solid-state thin film (red), shown alongside the laser excitation spectrum (gray) and the linear photocurrent response (green). The spectral overlap confirms that excitation predominantly addresses the lowest electronic transitions.

A photocell was obtained by depositing a colloidal CdSe QD solution in a tightly packed solid-state assembly on an interdigitated substrate. The nanocrystals in solution, functionalized with the native capping ligands (mainly trioctylphosphine oxide and hexadecylamine) exhibit a lowest-energy optical transition at approximately 16950 cm^–1^ (590 nm), as confirmed by the absorption spectrum (blue curve in Figure 2). This transition corresponds to an average QD diameter of about 3.7 nm with a size dispersion of σ = 8% in diameter, as verified by transmission electron microscopy (TEM; Figure S7), in agreement with ref 28 The absorption spectrum in solution displays two prominent low-energy bands conventionally assigned to the 1S_h_–1S_e_ (|1S⟩) and 2S_h_–1S_e_ (|2S⟩) transitions.^4,5^ The photocell device was fabricated by depositing via spin coating 10 layers of CdSe QDs onto the interdigitated gold electrodes (see Methods section). After the deposition of each QD layer, the native insulating long-chain alkyl ligands on the QD surface were replaced through a mass-action ligand-exchange process in solution with the short bifunctional ligand propanedithiol (pDT, chain length ≈ 0.55 nm). This ligand exchange simultaneously enables robust anchoring of the initial QD layer to the gold electrodes, enhances interparticle electronic coupling due to the short ligand length, and promotes sufficient electrical conductivity for photocell operation under an applied electrical bias.^1,5,13,29^ The absorption spectrum of the solid-state assembly deposited directly on the integrated device substrate (red curve in Figure 2) shows a red shift arising from the variation in the dielectric constant of the environment and the interdot interactions promoted in the assembled film.

Figure 2 also shows the spectral linear photoresponse of the photocell (green curve), extracted from the PC-2DES data using the phase modulation technique. This measurement is essentially equivalent to the standard spectral electrical response characterization of a photocell and supports the reliability of our phase modulation approach. A comparison between the absorption spectra and the linear PC response of the solid-state QDs film reveals an enhanced signal intensity in the low energy region (between 16100 and 16400 cm^–1^), below the first excited-state transition (see also Figure S5). This enhancement reflects the spectral overlap between the excitation laser profile and the sample absorption. Indeed, as in the previous study,^5^ the laser profile covers only the first excited state (|1S⟩) and it enhances the response from lower-energy states due to its spectral shape. This profile was deliberately chosen based on our previous findings that the possibility of isolating interdot electronic coherences from the more common intradot ones depends critically on the choice of excitation profile, favoring those that selectively cover only the |1S⟩ band.^5,7,12^ The impact of this choice on the observed dynamics will be further clarified in the analysis of the fourth-order response.

### 2.2 PC-2DES Characterization

Figure 3 reports PC-2DES maps (absorptive signal, calculated as the sum of the rephasing and nonrephasing contributions) at population times of 0, 100, 200, and 400 fs (rephasing, nonrephasing, and absorptive maps for additional population times are shown in the Supporting Information, Figure S3 and Figure S4). All maps clearly exhibit a square pattern with two features along the diagonal, accompanied by cross peaks at symmetric coordinates above and below the diagonal. As expected, the signal profile along the diagonal closely follows the linear PC response (green curve in Figure 2a), showing a first maximum at (16900, 16900) cm^–1^, falling at energies similar to the |1S⟩ transition, and a second maximum at (16200, 16200) cm^–1^, corresponding to the low-energy shoulder observed also in the linear PC spectrum. As noted previously for the linear PC response, the spectral profile of the excitation pulses is expected to enhance contributions from red states. Nonetheless, a first qualitative comparison between the absorptive 2DES maps recorded for similar samples under analogous experimental conditions, using PC and coherent optical detection (Figure S6), clearly shows that these red states contribute much more significantly to the PC response, while they give rise to almost no signal in the optical detection. It is worth noting that, unlike optical detection, PC-detected spectral features can only emerge if a specific transition changes photoconductivity. This distinction provides a plausible explanation for the enhanced visibility of red-shifted states in the PC-2DES response.

**Figure 3:**
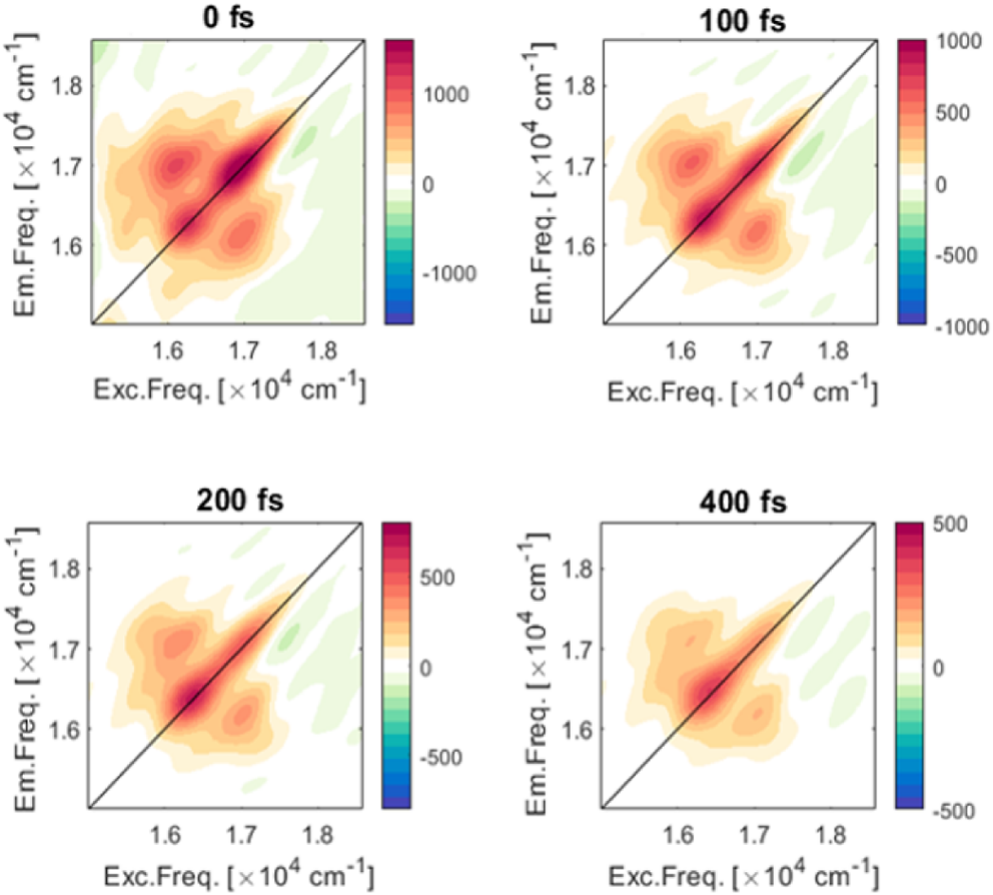
Fourth-order photocurrent response and population-dynamics analysis. PC-2DES absorptive maps at selected population times 0, 100, 200, and 400 fs. The spectra show two main diagonal features corresponding to the |1S⟩ transition and a red-shifted band, together with symmetric cross peaks indicating spectral correlations among these states. The elongated diagonal profile reflects inhomogeneous broadening, whereas the strong contribution of the red-shifted band highlights its dominant role in photocurrent generation.

The presence of cross peaks appearing at early population times between the two main diagonal peaks indicates spectral correlations among the states responsible for these signals. The interpretation of cross peaks in action-detected 2DES is particularly interesting and remains debated, as it is now clear that the conventional understanding from C-2DES cannot be directly applied to PC-2DES.^15^ While in C-2DES early time cross peaks reflect excitonic delocalization, in action-based 2DES they may also arise from other kinds of spectral correlations (including exciton–exciton annihilation^15,30–33^ and incoherent mixing^34,35^ ). In strongly coupled systems like our samples, however, cross peaks are intrinsic to the nonlinear response and probe the same excitonic states as in C-2DES.^36^

More insight can be obtained by examining the signals’ dynamic behavior. To gain a quantitative understanding of the time evolution, the dynamics along *t*_2_ were analyzed using a global multiexponential complex fitting approach.^37^ Instead of fitting individual kinetic traces at selected frequencies, global analysis employs a common set of time constants to simultaneously fit the dynamics across the entire 2D map. This procedure yields a series of time constants, with their associated amplitudes represented as a function of excitation and emission frequencies, in the form of 2D decay-associated spectra (2D-DAS). Each 2D-DAS identifies the spectral features that decay or rise with a specific time constant, thereby enabling the assignment of distinct relaxation pathways involving different states. Within the framework of the global fit, positive (negative) amplitudes in a DAS correspond to decaying (rising) spectral contributions associated with the corresponding time constant.^37^

The analysis applied to our data reveals, in addition to a constant component associated with processes occurring on a time scale much longer than the experimental window (>1 ps), three distinct dynamical components with time constants of approximately 10, 60, and 280 fs. The corresponding 2D-DAS are shown in Figure 4a–c. The quality of the global fit is demonstrated by the excellent agreement between the experimental data and the fitted curves for the kinetic traces at selected representative coordinates of the 2D maps, reported in Figure 4d–f. When interpreting the dynamical components in PC-2DES data, it should be kept in mind that the detected signal is a PC proportional to the population of the excited state generated in the sample after the fourth light–matter interaction, which has undergone an effective dissociation mechanism.^18^ Therefore, this signal will not account for all dynamic processes that, although they contribute to overall relaxation, do not lead to charge separation events.

**Figure 4:**
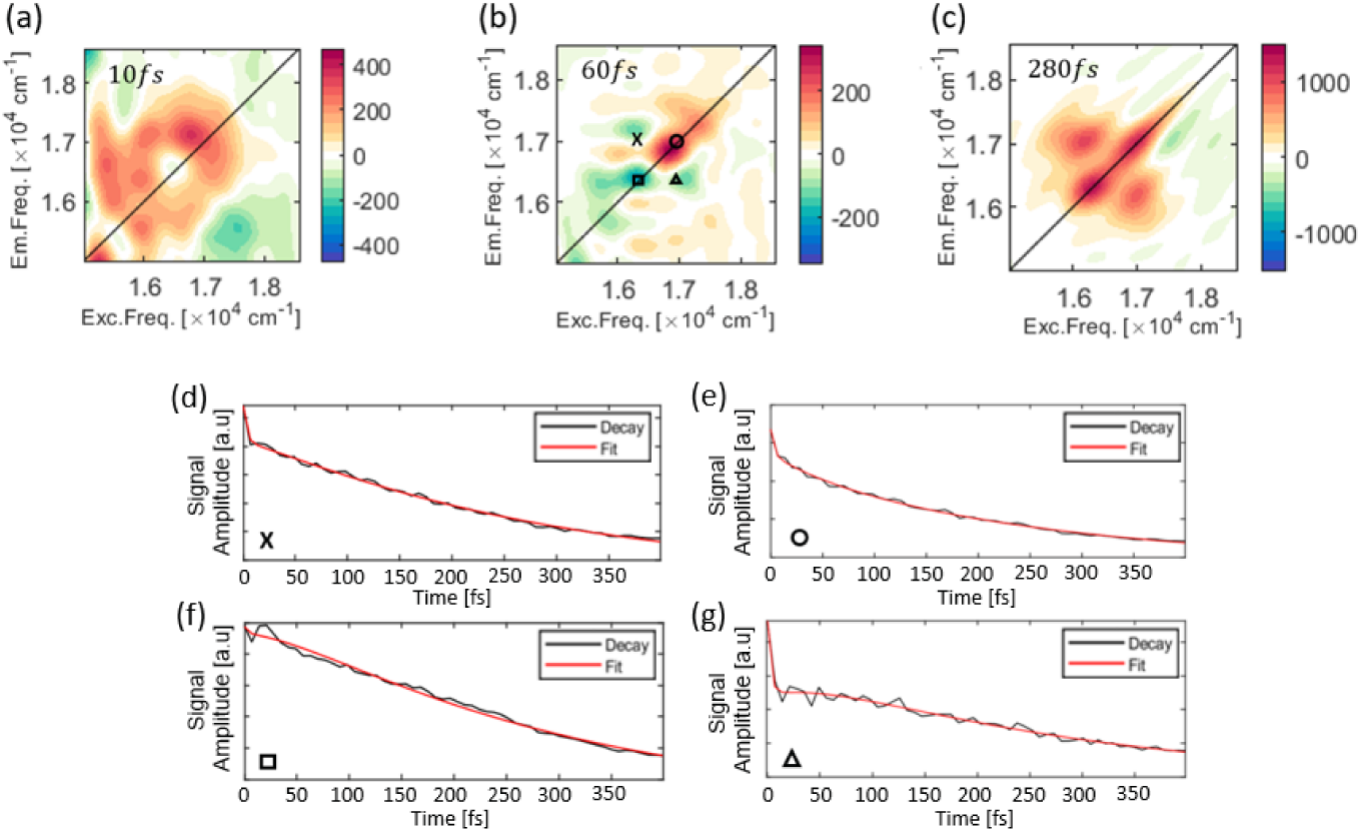
Population dynamics. 2D-DAS showing the amplitude distribution associated with the first three time constants, as obtained from the global fitting analysis: (a) 10 fs, (b) 60 fs and (c) 280 fs. (d–g) Example population-time traces extracted from selected spectral coordinates of the 2D maps (black lines), labeled by the corresponding markers in panel b, shown together with the global fit curves (red lines). These traces illustrate the distinct temporal behaviors of different spectral features across the *t*_2_ dimension.

The first component has a time constant of about 10 fs. Although this value is close to the time resolution of our experiment, it is worth noting that, unlike C-2DES, which is particularly prone to artifacts at early times due to solvent contributions, scattering, and pulse overlap effects, PC-2DES is ideally suited for measuring signals in the pulse-overlap region (around *t*_2_ = 0 fs), owing to its intrinsic phase stability and distinctive PC-based detection.^15,32,38,39^ The 2D-DAS of this component (Figure 4a) shows a decaying amplitude distributed across all diagonal and off-diagonal positions. Similar amplitude distributions observed in conventional C-2DES experiments on strongly coupled systems are typically attributed to the ultrafast dephasing of coherent superpositions of excitonic states instantaneously prepared by the excitation pulses.^40,41^ These superpositions dephase immediately after photoexcitation, evolving on a time scale comparable to the pulse duration.^40,41^ Interestingly, the observation of these dynamics in QD solids is typically inaccessible in C-2DES due to the strong scattering contributions that dominate the pulse-overlap region. By contrast, their visibility in PC-2DES indicates that coherent superpositions of excitonic states play a role in the very earliest stages of carrier generation, providing new insight into the mechanism of charge separation.

The second, and most interesting, component, with a time constant of 60 fs (Figure 4b), was not previously identified in C-2DES measurements. It exhibits a distinct 2D-DAS characterized by a strong positive (decaying) contribution at the upper diagonal peak (circle marker), accompanied by negative (rising) contributions slightly blue-shifted with respect to the lower diagonal peak (square marker) and at both cross-peak positions (cross and triangle markers). These negative amplitudes indicate the presence of a rising component within the global fit, which can also be appreciated in the kinetic traces shown in Figure 4d,f,g as a deviation from a purely monotonic decay. When interpreting the physical meaning of this component, it is important to recall that the PC-detected nature of the measurement ensures that the signal selectively reflects only those processes contributing to charge separation and transport. This suggests that the dominant contribution to the ∼60 fs component arises from mechanisms that are particularly effective at generating photocurrent, rather than from purely cooling processes. Therefore, this behavior reflects an ultrafast process involved in the early stages of carrier generation and transport, as discussed in the next section.

The third component (280 fs, Figure 4c) describes a global decay of all features in the 2D maps and therefore must correspond to a process that reduces the PC signal at every spectral coordinate. Decay dynamics on similar time scales have already been reported in previous ultrafast studies and likely arise from a combination of mechanisms, including electron–electron scattering and surface-related relaxation channels.^4,5,42,43^

### 2.3 Interpretation of the PC-2DES Response

Despite extensive study, the dominant transport pathways in QD solids, particularly in the subpicosecond regime, remain the subject of ongoing debate, as this ultrafast time scale is still largely unexplored. Carrier motion depends on a complex interplay among the Bohr radius, interdot spacing, and structural disorder of quantum dots. Moreover, when QD solids are integrated into devices, junctions with dissimilar materials introduce interfacial states that strongly influence carrier generation and extraction. As a result, assigning a specific microscopic process to the identified time constants is inherently challenging.^6^

Nevertheless, the spectral distribution of signals observed in the 2D maps, together with their temporal dynamics, provides valuable insight into carrier-transport mechanisms in these QD solids. The most salient feature is the dominant presence of red-shifted states, observed in both linear and nonlinear PC responses at approximately 16200 cm^–1^, which contribute only weakly to the corresponding optical signals. These states clearly correlate with the |1S⟩ transition (16900 cm^–1^), as demonstrated by the presence of off-diagonal peaks at cross coordinates (Figure 3), and they participate in an ultrafast process that leads to an increase in PC (Figure 4).

Several mechanisms could, in principle, account for the red-shifted signal observed at 16200 cm^–1^. First, the involvement of trap states can be excluded, as carrier trapping typically occurs on much longer time scales and is therefore inconsistent with the ultrafast dynamical changes observed here.^44–48^ We also considered whether the red shift could arise from a global shift of the energy levels induced by the Stark effect under the static bias applied for PC detection. The Stark shift was estimated by introducing a field-dependent term into the effective-mass Hamiltonian (see Supporting Information). In agreement with previous studies on similar materials,^49^ the bias-induced Stark shift was found to be significantly smaller than the red shift experimentally observed in both the linear and nonlinear PC responses. Also the contribution of biexcitons can likewise be excluded, as biexciton formation is strongly suppressed under our experimental conditions due to the very low excitation density, for which the average number of photogenerated carriers is ≪1. Furthermore, the spectral position and temporal dynamics of the 16200 cm^–1^ signal are incompatible with the characteristic behavior of biexcitons reported in extensive femtosecond time-resolved studies.^45,50,51^

In previous work, we developed a detailed excitonic model to describe the electronic structure and the ultrafast 2D electronic response of size-dispersed strongly coupled CdSe QDs in solid samples, analogous to those studied here. Using a k·p effective-mass Hamiltonian that incorporates Coulomb, spin–orbit, and crystal-field interactions, the fine-structure excitonic levels of isolated QDs and their dimers were built.^11^ To properly account for ensemble inhomogeneity, a Liouvillian ensemble-averaged method was introduced, capturing the dominant Gaussian broadening due to size variation. The key outcome is the formation of a new set of eigenstates, significantly delocalized over more dots, some of them red-shifted with respect to the |1S⟩ transition of monomeric QDs.^7,12^ These predictions were also experimentally validated by C-2DES.^5^

These red-shifted, delocalized states may also explain the appearance of the low diagonal peak in PC 2D maps. The delocalized, red-shifted bands have relatively weak oscillator strengths and therefore do not correspond to particularly bright optical transitions.^5^ However, they have been proposed to enhance photocurrent by facilitating long-range charge separation beyond the Coulomb capture radius,^52–54^ and through the formation of trions.^55,56^

Trions are charged three-body states consisting of an exciton bound to an additional electron or hole that, depending on the sign of the additional charge, can thus be either positive or negative.^57–60^ The characterization of the formation and relaxation dynamics of trions is attracting increasing interest in optoelectronics because their properties are gate-tunable, allowing electrical control over light-matter interactions, and they enable the conversion of optical signals into electrical currents (optoelectronic conversion).^61^ Trions are more easily formed when excitons are delocalized because a larger extension of the exciton increases the probability for this exciton to interact with residual background carriers, resulting in a faster trion formation.^55,56^ Excitons and trions are also expected to be spectrally correlated, with cross-diagonal peaks revealing exciton–trion coupling and conversion processes,^55^ as also observed in our PC-2DES maps. The formation of trions and their spectral correlations with (delocalized) excitons might explain the peculiar signal distribution in the 2D-DAS of the ∼60 fs time component and justify the ultrafast increase of the PC signal at the lower diagonal peak and cross-peaks positions (Figure 4b).

Trion formation typically occurs when excitons interact with excess charges, which, in our device, are most plausibly electrons injected under the applied bias used to promote exciton dissociation. Indeed, a simple electrostatic estimate yields a charging energy of approximately 70 meV for a ∼3.7 nm CdSe QD, while a 10 V bias applied across a 300 nm gap corresponds to an energy scale of roughly 120 meV per elementary charge over the dot diameter (Section S5, Supporting Information). This supports the plausibility that the applied bias can inject and/or redistribute electrons and promote coupling between states. In the presence of residual free electrons, photoexcited excitons may bind with these excess charges to form negatively charged excitons.

Previous studies of trions in CdSe nanocrystals have relied primarily on low-temperature time-resolved photoluminescence spectroscopy to probe their radiative decay.^62–64^ However, the ultrafast formation times of trions have so far remained experimentally inaccessible. Despite this, both their formation and subsequent relaxation dynamics are known to play a central role in determining the performance of PC-based devices under operating conditions.^63^ Moreover, supporting this interpretation, recent transient absorption experiments performed under electrochemical control^63^ have reported hot-electron relaxation in trionic states with a time constant of about 370 fs, attributed to electron–electron scattering. This value is remarkably close to the 280 fs time constant of our third dynamical component, further reinforcing the assignment.

### 2.4 Beating Analysis

An additional important indication emerging from the global fit concerns the presence of coherent dynamical mechanisms active during the relaxation dynamics, manifested as beatings of the signal amplitudes at specific coordinates as a function of the population time *t*_2_. In our previous C-2DES measurements, we have already characterized the coherent dynamics of this material, identifying several beating frequencies corresponding to different coherent dynamic phenomena. Beyond the well-known longitudinal optical (LO) phonon consistently observed at about 230 cm^–1^ in several time-resolved experiments,^5,65–67^ several rapidly damped high-frequency modes were detected and attributed to intra- or interdot coherences based on theoretical simulations.^5,11^

Figure 5 qualitatively compares the time–frequency transform (TFT) analysis performed at a representative cross-peak position for the C-2DES and PC-2DES data. TFT analysis overcomes the limitations of conventional Fourier-transform-based approaches by preserving both frequency and time resolution. In a TFT spectrum, the ordinate reports the frequencies of the components contributing to the beating pattern at a specific coordinate of the 2D map, while the abscissa represents their temporal evolution.^68,69^ The first emerging observation is that, while the TFT map from C-2DES data is dominated by the LO beating, in PC detection this feature is completely absent, as shown in the signal traces in Figures 5c,d. The suppression of the 230 cm^–1^ mode with PC detection suggests that the LO phonon, although it strongly affects the optical response, plays a negligible role in PC generation. This finding is important not only as an indication of the carrier transport mechanism in QD solids, but also because it enables clearer detection of contributions from electronic coherences, which in optical detection are partly obscured by the dominant low-frequency LO beatings.

**Figure 5:**
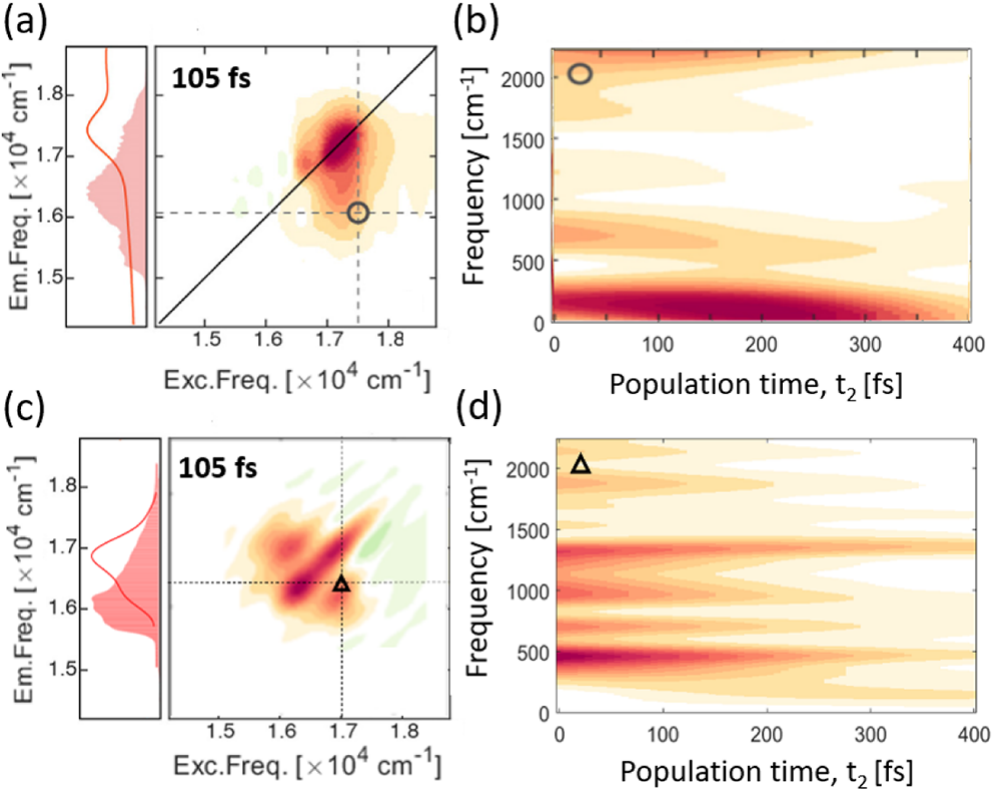
Comparison of the coherent beating behavior observed in C-2DES (a, b) and PC-2DES (c, d). (a) C-2DES map at 105 fs. The left panel shows the absorption spectrum (line) overlaid with the pulse profile (red area). (b) TFT analysis at the below-diagonal cross-peak coordinates (indicated by the circle marker), highlighting the dominant contribution of LO phonon beatings at 230 cm ^–1^. (c) PC-2DES map at 105 fs. The left panel shows the linear PC response overlaid with the pulse profile. (d) TFT analysis at the below-diagonal cross-peak coordinates (indicated by the triangle marker); no contribution from the LO phonon is detected.

Analysis of the beatings in the PC-2DES data reveals relatively strong features around 500, 700, 900, and 1300 cm^–1^ (Figure 5d), which primarily contribute at the cross-peak coordinates and are characterized by short dephasing times. While a fully quantitative comparison is not meaningful owing to slight differences in the samples and experimental conditions, as well as possible effects associated with the different detection schemes, the signals observed in the PC-2DES data (Figure 5d) are in excellent qualitative agreement with those found in the C-2DES measurements (Figure 5b), both in terms of frequency range and dephasing dynamics, suggesting a likely common origin. In particular, modes with these frequencies in C-2DES have previously been attributed to interdot electronic coherences, involving different combinations of superpositions between states belonging to the lowest energy manifolds.^11^ These results confirm that laser-induced electronic coherences, already known to contribute to the optical response, also contribute to PC generation, at least in the earliest ultrafast time window, whereas vibrational coherences do not. Moreover, the interdot nature of the beatings supports our hypothesis that PC is generated primarily through delocalized interdot excitonic manifolds.

## 3 Conclusions

In conclusion, PC-2DES reveals that charge generation in CdSe QD solids on ultrafast time scales is governed by delocalized excitonic states. We demonstrate that PC-2DES directly links coherent excitonic dynamics to measurable charge generation in a functioning CdSe QD photocell. By selectively exciting the |1S⟩ manifold, we isolate low-energy delocalized states that dominate the PC response despite contributing only weakly to the optical response. Global kinetic analysis uncovers three dynamical components, whose interpretation is summarized in Figure 6. After the initial decay of the PC signal due to dephasing and ultrafast relaxation events, happening in a time scale comparable with pulse duration (∼10 fs), an increase of the PC signal at specific spectral coordinates with a time scale of (∼60 fs) is recorded, compatible with possible trion formation under applied bias. At longer times (∼280 fs), the dynamics are dominated by carrier relaxation and transport through the quantum dot film toward the electrodes, accompanied by competing recombination processes that reduce the overall signal. Importantly, the PC-detected signal selectively reflects only those pathways that lead to charge separation and transport, providing a direct link between the observed spectral features and the underlying charge-generation mechanisms. Moreover, in contrast to optical detection, PC-2DES suppresses LO-phonon signatures, enabling the clearer observation of higher-frequency electronic coherences attributable to interdot coupling.

**Figure 6:**
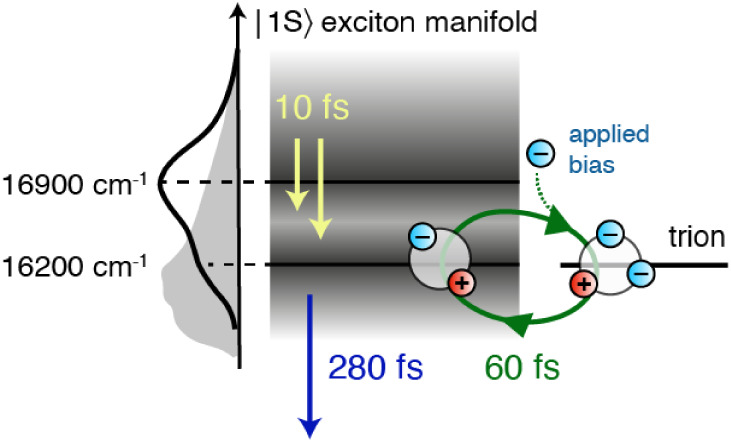
Schematic illustration of the processes contributing to the PC-2DES signal. The left panel shows the linear PC response (black line) overlaid with the pulse profile (gray area).

These findings demonstrate that PC-2DES can probe coherent charge-generation pathways under true device-operating conditions. By providing direct mechanistic insight into how excitonic coherence and delocalization contribute to PC generation, this approach provides a framework for coherence-informed design principles in next-generation quantum-dot optoelectronic devices.

## 4 Methods

### 4.1 Sample Preparation of CdSe Colloidal QDs

The QDs were synthesized following a previously reported procedure.^70,71^ Briefly, 1 mmol of CdO dissolved in 1 mL of oleic acid was heated at 90 °C to form the Cd-oleate complex. 23 mmol of trioctylphosphine oxide and 37 mmol of hexadecylamine were transferred into the Cd oleate flask. The temperature was then increased up to 300 °C and the Se precursor solution (5 mmol dissolved in 4.5 mL of tributylphosphine) was injected at 295 °C. The temperature was then reduced at 270 °C and kept constant for 90 s, to allow the nucleation and growth of the colloidal CdSe nanocrystals. The QDs were first purified, then collected by adding ethanol and redispersed in 4 mL of hexane, resulting in concentration of 2·10^–4^ M.

### 4.2 Photocell Preparation

Twenty photocells were prepared on 1 × 1 cm quartz substrate. First, each substrate contains 80 lithographically designed interdigitated gold electrodes fabricated by ConScience. Each device consists of source-drain electrodes in an interdigitated structure with 100 set of electrodes; each finger has a length of 34 μm, a width 1.2 μm and a gap of 300 nm between fingers (Figure S8a). The photoactive thin film was fabricated by sequentially depositing ten layers of CdSe quantum dots (QDs) onto the patterned interdigitated substrate via spin coating. Prior to QD deposition, the substrate was cleaned by alternating sonication in acetone and isopropanol for two cycles, followed by drying under a gentle nitrogen flow. The cleaned substrate was subsequently immersed for 10 min in an ethanolic solution of propanedithiol (pDT) with a concentration of 4.5 × 10^–5^ M to promote uniform functionalization of the gold electrodes and facilitate QD attachment. Each QD layer was deposited by spin coating 20 μL of a concentrated colloidal CdSe QD solution in hexane at 1000 rpm for 30 s. Following the deposition of each layer, the substrate was immersed in the pDT solution for 10 min to induce ligand exchange, replacing the native long-chain ligands (trioctylphosphine oxide, oleic acid, and hexadecylamine) with the short bifunctional pDT ligands. All fabrication steps were performed under ambient conditions at room temperature. After deposition, the chips were mounted onto a leadless chip carrier (LCC), and source-drain pads of 20 devices were wire-bonded to the LCC pads. The LCC was inserted into a sealed Dual In-line Package (DIP)-to-LCC carrier, and a glass slide was attached under inert atmosphere for encapsulation (Figure S8b).

### 4.3 PC-2DES Measurements

A 3 kHz Ti:Sa amplified laser system (Coherent Libra) was used to pump a home-modified noncollinear optical parametric amplifier (TOPAS-white). The resulting broadband beam passed through an acousto-optic pulse shaper (Fastlite Dazzler), which generated, from each laser shot, a sequence of four collinear time-delayed pulses with independently controlled phases. A prism compressor was employed to optimize the temporal resolution, and an autocorrelation measurement yielded a pulse duration of approximately 10 fs. The beam was focused to a 1 mm waist on the CdSe photocell.

During the measurements, a d.c. bias voltage of 10 V (∼3.3 × 10^7^ V/m) was applied to the device via the bias input of a FEMTO Messtechnik GmbH DLPCA-200 low-noise current amplifier. The resulting d.c. photocurrent was amplified by the DLPCA-200 and acquired using a National Instruments NI USB-4432 data acquisition board. This voltage was optimized to ensure a sufficiently strong photocurrent signal while avoiding device degradation or electrical shunting. The excitation intensity was similarly optimized to prevent many-particle interactions. Reference measurements performed on a device lacking the QD thin film produced no detectable signal, confirming that the recorded response originates from the quantum dot layer.

### 4.4 Phase Modulation

A phase-modulation scheme was implemented by varying the phases of each pulse in the sequence according to a specific pattern that enables phase-locking throughout the experiment and allows digital lock-in detection. Thanks to the storage capabilities of the system, we generated 72 distinct phase patterns for each sequence. For every fixed set of delays *t*_1_, *t*_2_, and *t*_3_, the pulse phases were varied as follows: the phase of the first pulse was kept constant (ϕ1 = 0), while the phases of pulses 2, 3, and 4 were incremented at each laser shot by π/6, π/8, and π/9, respectively. Consequently, the 73rd laser shot resets to the first pattern of the next sequence. This cycle was repeated 42 times, yielding 42 sequences for each set of fixed delays.

The resulting modulation frequencies are therefore *f*_1_ = 0, *f*_2_ = 6 ×42 = 252 Hz, *f*_3_ = 8 × 42 = 336 Hz, *f*_4_ = 9 × 42 = 378 Hz. To extract the fourth-order signal, the detected output was digitally filtered at the appropriate frequency combination. For example, the rephasing contribution appears at *f*_reph_ = −*f*_1_ + *f*_2_ + *f*_3_ – *f*_4_ = 210 Hz while the linear contribution was isolated at *f*_linear_ = *f*_3_ + *f*_4_ = 588 Hz.

## Supplementary Material



## Data Availability

The data that support the findings of this study are available from the corresponding author, E.C., upon reasonable request.

## Notes

Additional experimental methods and results; supplementary PC-2DES maps; photocurrent and device characterization; TEM analysis; Stark-shift calculations; further details of the theoretical calculations (PDF)
