# Supplementary material for: Probing Ultrafast Excitonic Coherences and Charge-Generation Pathways in Quantum-Dot Photocells via Photocurrent-Detected Two-Dimensional Electronic Spectroscopy
Source: ACS Nano. 2026 Jun 22;20(26):18863–72. doi: 10.1021/acsnano.6c04829 (PMC13348165; doi:10.1021/acsnano.6c04829)
Supplement: Supplementary file 1 [file nn6c04829_si_001.pdf]

SUPPORTING INFORMATION

Probing Ultrafast Excitonic Coherences and Charge-Generation Pathways in Quantum-Dot Photocells via Photocurrent-Detected Two-Dimensional Electronic Spectroscopy

Hadar Manis Levy[1], Matilde Doardo[1], James R. Hamilton[2,5], Carlo Nazareno Dibenedetto[3], Thibault Degousée[4], Jan A. Mol[4], Marinella Striccoli[3], Francoise Remacle[2,5], Elisabetta Collini[1]

[1] *Dipartimento di Scienze Chimiche, Università degli Studi di Padova, Padova 35131, Italy*

[2] *Theoretical Physical Chemistry, Research Unit Molecular Systems, University of Liege, B4000 Liege, Belgium*

[3] *CNR-IPCF SS Bari, c/o Chemistry Department, University of Bari Aldo Moro, Via Orabona 4, 70126 Bari, Italy*

[4] *School of Physical and Chemical Sciences, Queen Mary University, London E1 4NS, U.K.*

[5] *The Fritz Haber Center for Molecular Dynamics and Institute of Chemistry, The Hebrew University of Jerusalem, 91904 Jerusalem, Israel*

S1. Additional details about PC-2DES characterization

S1.1 Dazzler non-linearity artifact correction

A well-known artifact can arise in the photocurrent-detected two-dimensional spectroscopy (PC-2DES) technique when the photocurrent generated by the device is too low. In such cases, non-linearities in the setup components may obscure the device's intrinsic non-linear response and therefore must be corrected. In this study, the effect of pulse-shaper nonlinearity was corrected in post-processing by rescaling the measured signal using a correction factor obtained from independent photodiode measurements of the pulse sequence acquired in both the linear and nonlinear operating regimes of the pulse shaper. This procedure compensates for amplitude distortions introduced by the non-ideal response of the acousto-optic modulator. Figure S1a shows the rephasing time-domain manifestation of the non-linear artifact as measured with the linear photodiode. The device response before

and after applying the correction is shown in Figures S1b and S1c, respectively. Figures S1d and S1e display the absorptive response of the device before and after the correction.

While residual contributions from the excitation profile or early-time scattering cannot be entirely excluded, the structured spectral features and their well-defined temporal evolution indicate that the observed signal is dominated by intrinsic sample dynamics rather than experimental artifacts.

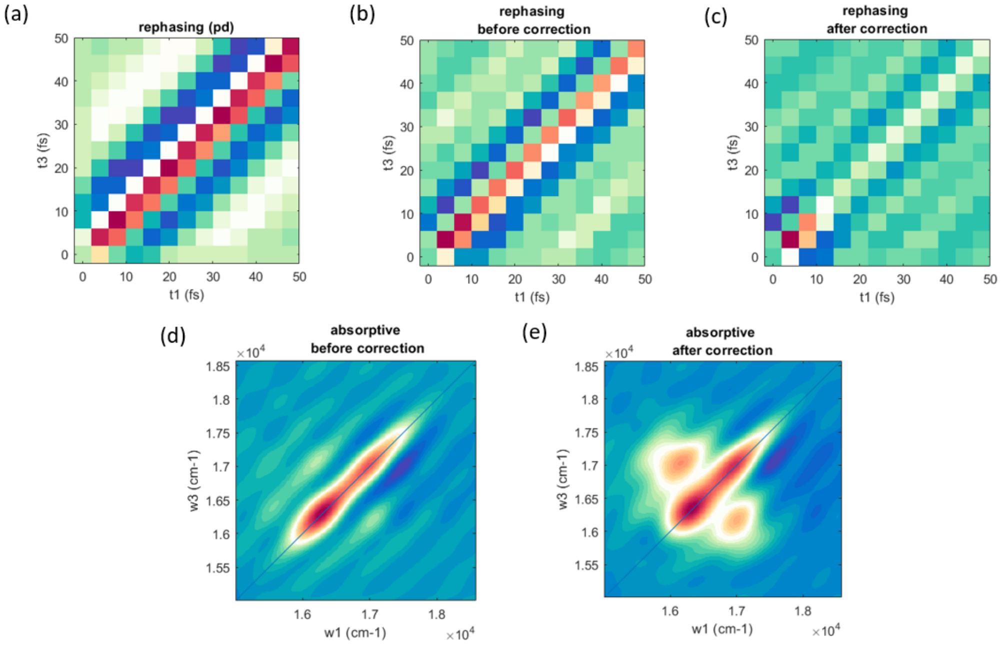

Figure S1. Correction of Dazzler-induced non-linearity in PC-2DES measurements. *(a) Time-domain rephasing trace showing the non-linear artifact measured using a linear photodiode. (b) Raw rephasing signal of the device before correction. (c) Corrected rephasing signal. (d) Absorptive spectrum before correction. (e) Absorptive spectrum after correction, showing removal of the artifact and recovery of the device's intrinsic response.*

S1.2 Verification of Linear Excitation Regime

To verify that the measurements were performed in the linear excitation regime, we measured the device signal as a function of pump pulse energy. Figure S2 shows the device signal as a function of pulse energy. The device signal corresponds to the output voltage of the current preamplifier, which is proportional to the current generated in the device under optical excitation. At low pulse energies, the device signal exhibits a linear dependence on excitation intensity, indicating that the response is dominated by single-excitation processes. At higher pulse energies, the signal progressively deviates from linearity, consistent with the

onset of saturation effects. The pulse energy used for all PC-2DES measurements (90 nJ, indicated by the arrow) lies within the linear regime.

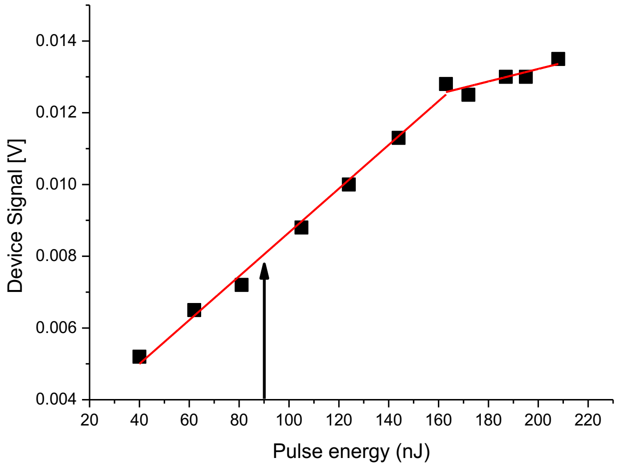

Figure S2. Device signal as a function of pump pulse energy. *The device signal shows a linear dependence on pulse energy at low excitation and deviates from linearity at higher pulse energies, indicating the onset of saturation. The pulse energy used for all PC-2DES measurements (90 nJ) is indicated by the arrow and lies within the linear regime.*

Exclusion of many-body interaction effects. We exclude any significant contribution from many-body particle interactions to the carrier dynamics because all experiments were performed at low excitation densities corresponding to $N_{eh} < 1$, where N_{eh} is the average number of electron–hole pairs excited per QD. N_{eh} was calculated as $N_{eh} = j_p \, \sigma_a$, with j_p the pump fluence (in photons cm^{-2}) and σ_a the QD absorption cross section. The pulse energy density was $\sim 1.15 \times 10^{-5}$ J cm^{-2} (corresponding to a 90 nJ pulse focused to a 1 mm diameter spot). Considering the laser spectrum between 550 and 650 nm, the resulting fluence is $\sim 3.5 \times 10^{13}$ photons cm^{-2}. The absorption cross section, determined following ref. [1], lies in the range $(0.2–10) \times 10^{-15}$ cm^2, yielding $N_{eh} \approx 0.2$, well below unity for all measurements.

S1.3 Additional 2DES maps

Figures S3 and S4 present PC-2DES maps at selected values of t_2 in the range 0-50 fs and 100-400 fs, respectively. These maps were collected by scanning t_1 and t_3 from 0 to 48 fs in steps of 4 fs, and t_2 from 0 to 400 fs in steps of 7 fs.

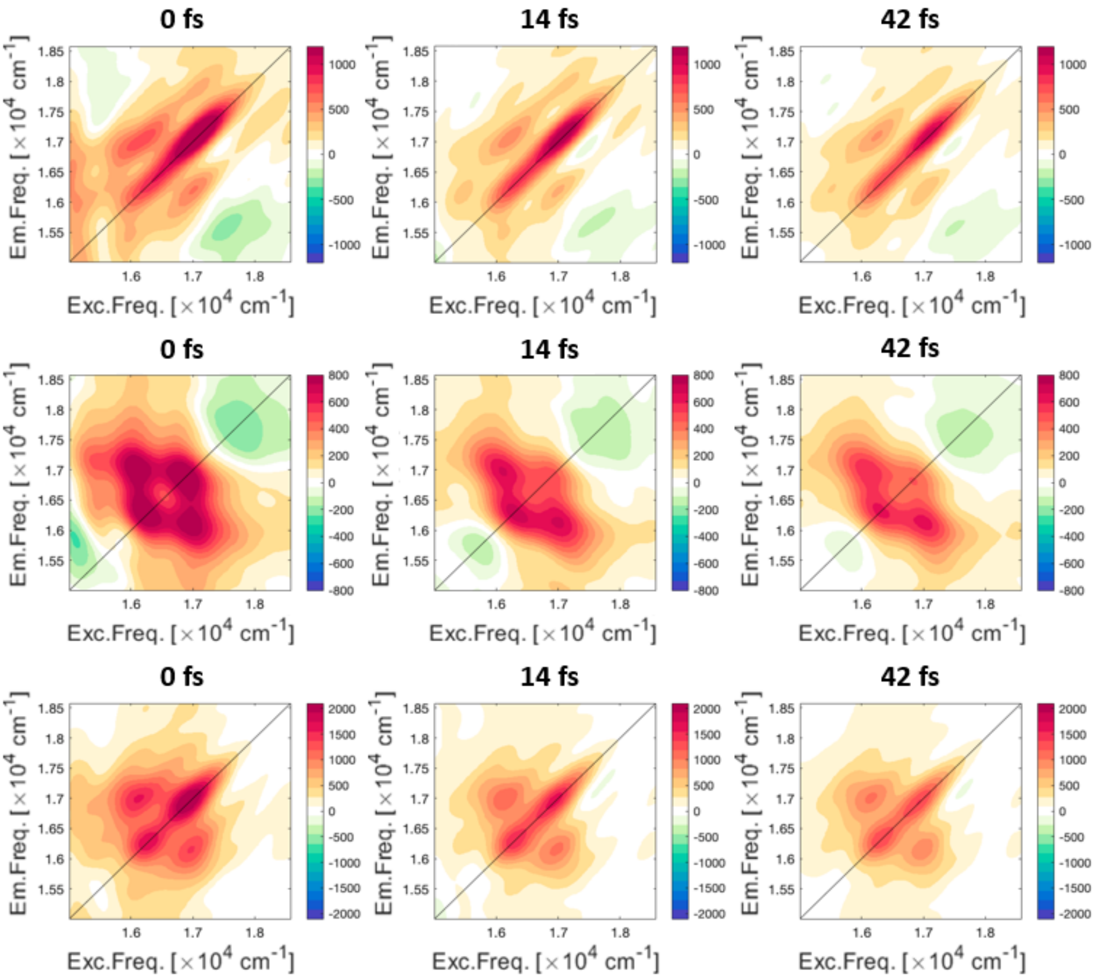

Figure S3. *PC-2DES maps for the rephasing (upper panel), non-rephasing (central panel) and absorptive contribution (lower panel) evaluated at short* t_2 *times. The intensity of the signal was normalized with respect to the map at 0 fs.*

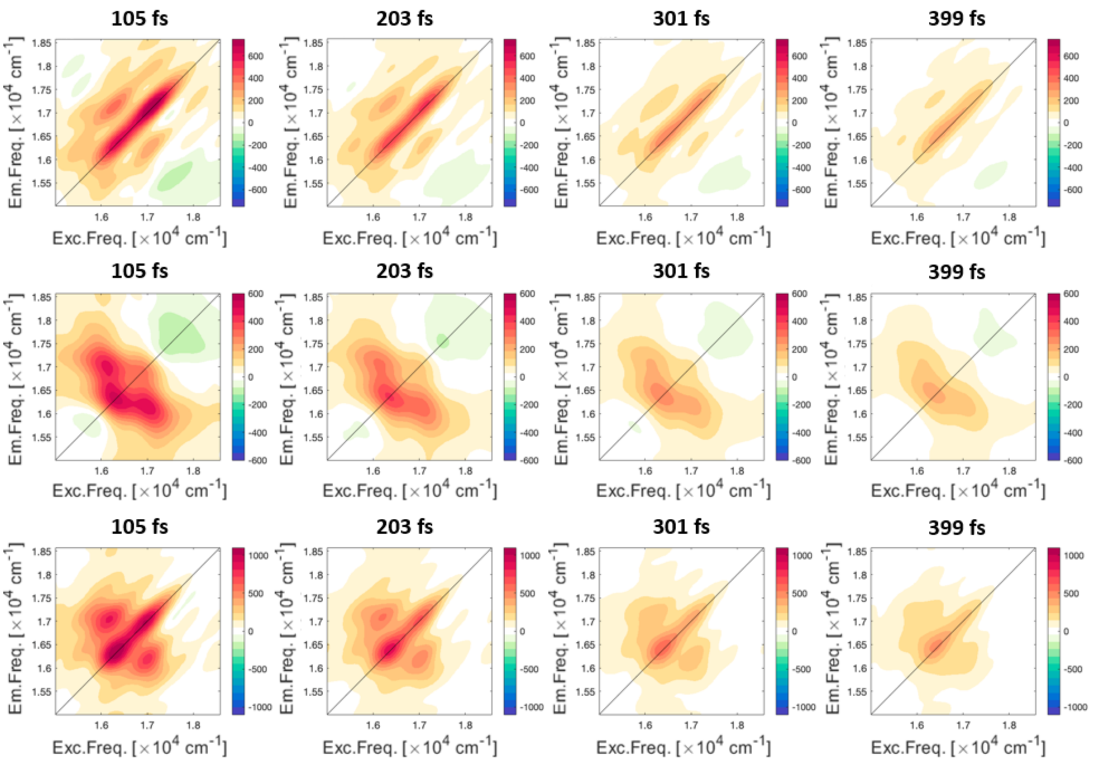

***Figure S4**. PC-2DES maps for the rephasing (upper panel), non-rephasing (central panel) and absorptive contribution (lower panel) evaluated at long t_2 times. The intensity of the signal was normalized with respect to the map recorded at 105 fs.*

S1.4 Photocurrent Response under Spectrally Filtered White-Light Illumination

To further support the interpretation of the enhanced contribution of red-shifted states to the PC response, we performed independent measurements of the spectral PC responsivity of the device under continuous white-light (WL) illumination. The incident light was filtered using a set of long-pass filters with cutoff wavelengths at 540, 603, 656, and 689 nm, thereby progressively removing higher-energy (shorter-wavelength) photons and selectively exciting the sample with lower-energy (redder) components of the spectrum. The measurements were carried out under an applied bias of 10 V, consistent with the conditions used in the PC-2DES experiments.

The resulting PC signal reflects the integrated response of the device to photons with wavelengths longer (i.e., lower energies) than the cutoff defined by each filter. The measured PC response as a function of the corresponding cutoff wavenumber is shown in Figure S5, together with the laser spectral profile used in the PC-2DES experiments and the absorption spectrum of the quantum dot thin film.

Despite the progressive reduction of the available excitation bandwidth and the weak optical absorption in the red region, a significant PC signal persists even when only the lowest-energy portion of the spectrum is transmitted. This contrast is particularly evident in the region highlighted by the blue arrow in Figure S5, where a measurable photocurrent response is observed despite the negligible optical absorption.

These observations indicate that red-shifted states, although weakly absorbing in linear optical measurements, can contribute efficiently to photocurrent generation. This behavior supports the interpretation that such red-shifted ("almost dark") states play a disproportionate role in charge generation and transport, consistent with the enhanced signal observed in the PC-2DES measurements.

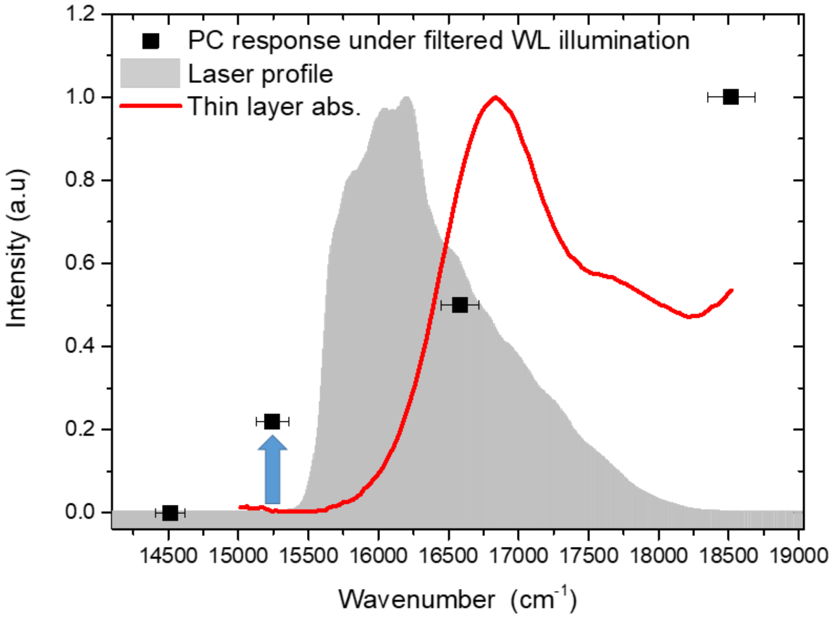

***Figure S5**. Photocurrent response of the quantum dot photocell under white-light illumination filtered with long-pass filters (black squares), plotted as a function of the corresponding cutoff wavenumber. Each point represents the integrated PC signal obtained for photons with wavelengths longer (i.e., lower energies) than the indicated cutoff. The gray shaded area shows the laser spectral profile used in the PC-2DES experiments, and the red line corresponds to the optical absorption spectrum of the thin film. The blue arrow highlights a spectral region where the photocurrent remains finite despite negligible optical absorption, indicating that red-shifted states contribute efficiently to charge generation.*

S1.5 Comparison of Optically Detected and Photocurrent-Detected 2DES Maps

To highlight the different detection selectivity of optical versus photocurrent readout, we report in Figure S6 a direct comparison between absorptive 2DES spectra recorded under identical excitation conditions using optical and PC detection. We note that the

measurements were performed on samples from different batches, with slightly different size distributions (average diameters of ~3.5 nm for the optically detected measurements and ~3.7 nm in the present study, see Figure S7), and under non-identical excitation conditions. The corresponding absorption spectra and laser excitation profiles are shown in Figure S6 to facilitate this comparison. Therefore, the comparison is intended to be qualitative.

In previous optically detected 2DES measurements, very early-time maps ($t_2 \lesssim 20$ fs) were reported, though not analyzed, as the optical signal is strongly affected by scattering and other coherent background artifacts that distort the spectra at short delay times. These effects hinder the extraction of reliable peak shapes and amplitudes. PC-2DES does not suffer from these early-time optical artifacts, since only population-related processes that modify the sample photoconductivity contribute to the detected signal. Consequently, PC-2DES permits meaningful analysis of 2DES maps even at ultrashort population times, such as the 14-fs maps shown in Figure S6.

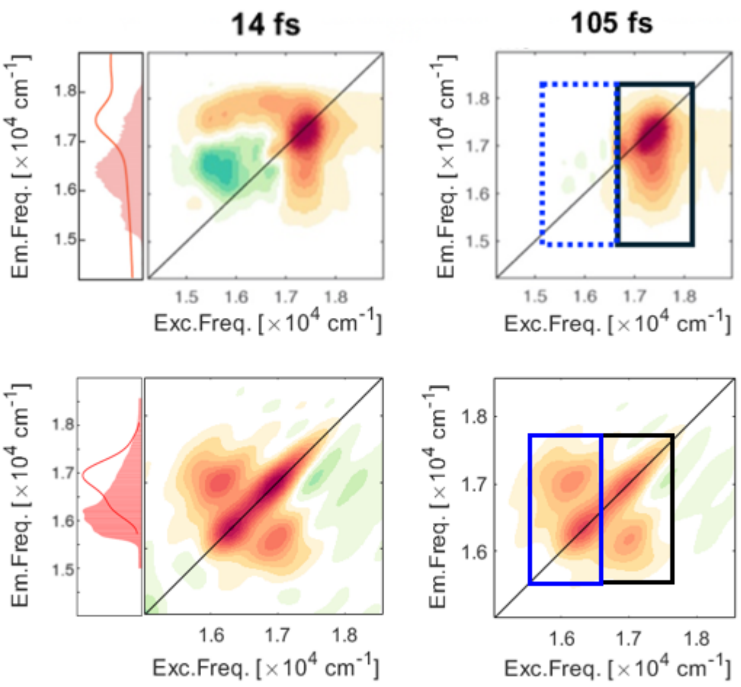

Figure S6. *Qualitative comparison between 2DES maps at two selected population times (t_2), obtained via optical detection (upper row) and PC detection (lower row). The two sets of experiments were performed on similar samples under analogous experimental conditions; the corresponding laser spectra and absorption profiles are shown for reference. The optically detected maps are reproduced from our previous work on multilayer CdSe QD samples.*[2]

S2. Transmission Electron Microscopy Characterization

Transmission electron microscopy (TEM) was used to determine the size and size dispersion of the CdSe quantum dots employed in this study. Representative TEM images show a dense ensemble of nearly spherical quantum dots with good size uniformity (Figure S7a). Individual particle diameters were extracted by analyzing several hundred dots from multiple regions of the sample to ensure statistical relevance. The resulting size distribution is shown in Figure S7b. The histogram is well described by a Gaussian function, yielding an average quantum-dot diameter of 3.7 nm with a relative size dispersion of $\sigma \approx 8\%$. These values are consistent with the optical transition energies observed in the linear absorption spectra discussed in the main text, confirming that the measured excitonic transition originates from quantum dots of the expected size.

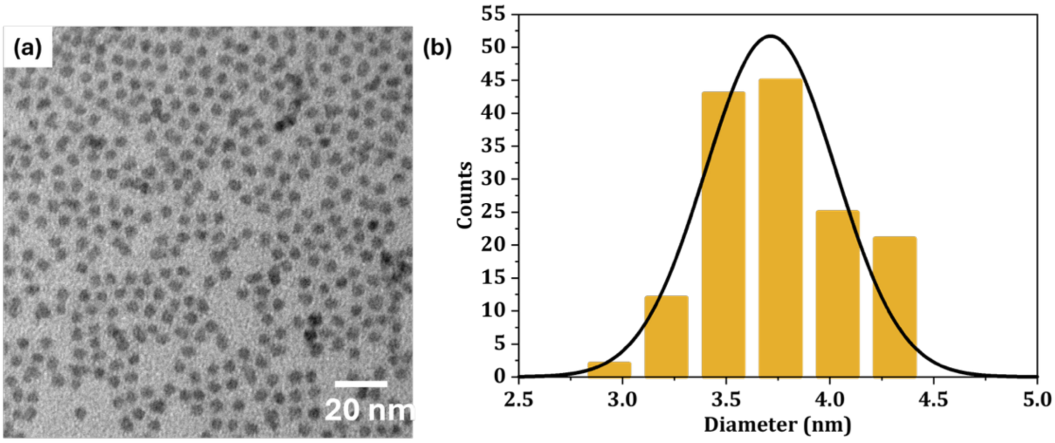

Figure S7. *(a) Representative transmission electron microscopy (TEM) image of the CdSe quantum dots used in this work, showing a dense and relatively monodisperse ensemble. (b) Histogram of quantum-dot diameters extracted from TEM images. The solid black line represents a Gaussian fit, yielding an average diameter of 3.7 nm with a size dispersion of σ ≈ 8%.*

S3. Device Architecture and Electrical Characterization

The device structure used in this study is shown in Figure S8a. A 1X1 cm quartz substrate contains 80 devices with interdigitated electrodes (100 electrode pairs per device; finger length 34 µm, width 1.2 µm, and inter-finger gap 300 nm). After deposition of the CdSe quantum dots, 20 devices were wire-bonded to a leadless chip carrier (LCC) and encapsulated under a N_2 atmosphere using a sealed Dual In-line Package (DIP)-to-LCC carrier (Figure S8b).

Current–voltage (I-V) measurements were performed in the ±10 V range under white light illumination and in the dark. Illumination results in a pronounced increase in the measured current across the full voltage range, whereas under dark conditions, the current is reduced by approximately three orders of magnitude (Figures S8c,d). Notably, the dark I-V response differs from that of an empty device (Figure S8d).

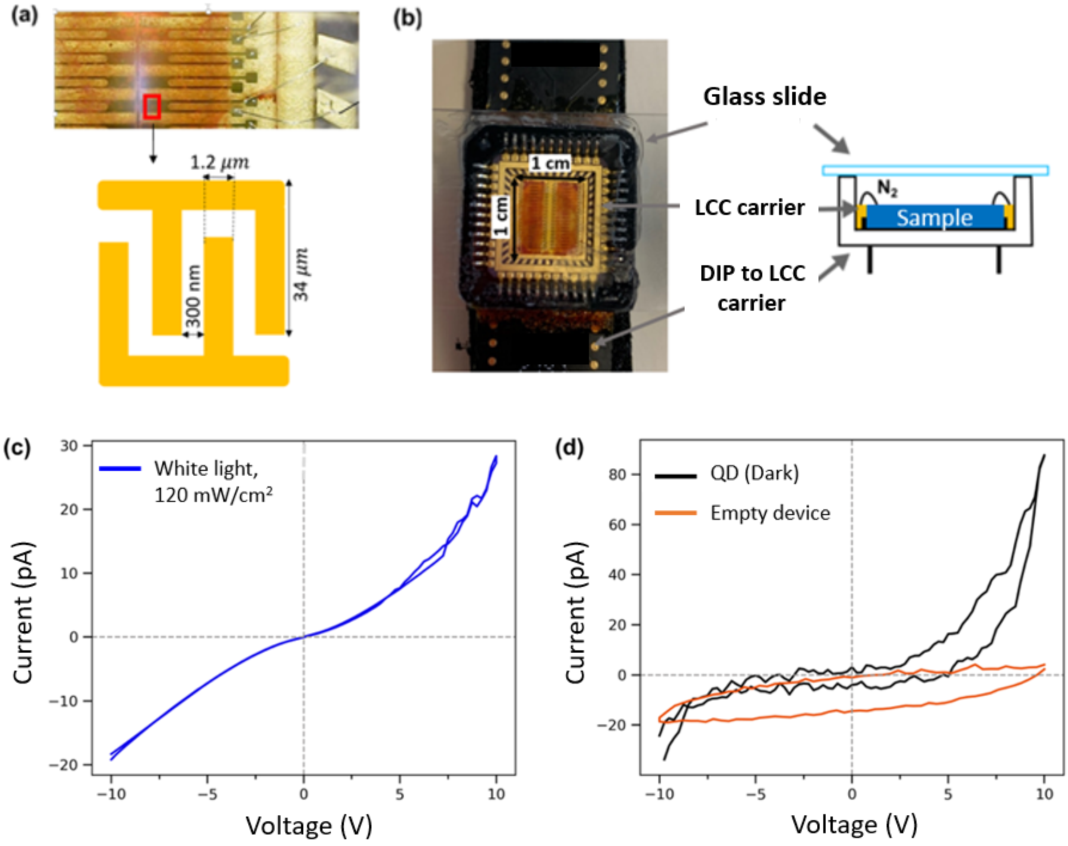

Figure S8. *(a) Optical microscope images of 8 devices; the inset shows a portion of the interdigitated structure of the electrodes. (b) Image and schematic of the encapsulated sample. (c) I-V curve of an encapsulated device under white light illumination. (d) I-V curve of and empty device (red) and of an encapsulated device in the dark (black).*

S4. The effect of the applied bias on the energies of the single QD fine structure states

The monomer is modeled as consisting of a ground state and two exciton states ($1S$ and $2S$). Each exciton state contains 12 fine structure states (FS) and so the system has a total of $N = 24$ FS states. [3,4] Figure S9 shows the field-free transition energies of these 24 FS states as a function of the ensemble-averaged monomer diameter ($\overline{D}$). The bands are

denoted by different colours: $1S_{3/2}$; $1S_{1/2}$; $2S_{3/2}$; $2S_{1/2}$. Figure S9 also shows that for $\overline{D} \gtrsim 3.5\ nm$ there is mixing of the $1S_{1/2}$ and $2S_{3/2}$ bands.

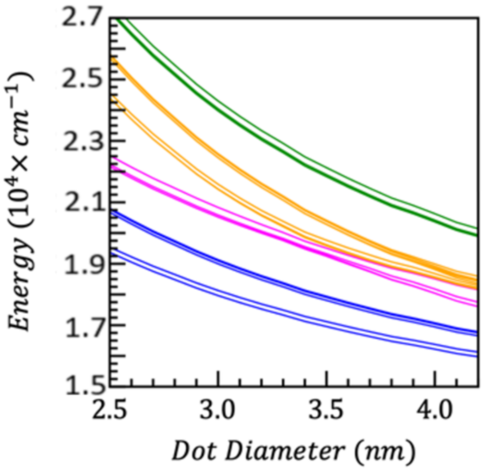

Figure S9. *Field free transition energies of the 24 FS states in the monomer as a function of the monomer ensemble average diameter* ($\overline{D}$). *The bands are denoted by different colors:* $1S_{3/2}$ $(blue)$; $1S_{1/2}$ $(pink)$; $2S_{3/1}$ $(yellow)$; $2S_{1/2}$ $(green)$.

The fine structure energy levels of the monomer can then be used to build the energies of a dimer, as described in refs. [3,4] The effect of the static field, $\mathcal{E}$, resulting from the application of the bias is included in the Hamiltonian in the dipole approximation:

$$\boldsymbol{H}(\varepsilon) = \boldsymbol{H_0} - \mathcal{E}\boldsymbol{\mu}$$

$\boldsymbol{H_0}$ is an $N \times N$ diagonal matrix. The diagonal elements are the bias free excited state energies, $\{E_i\}$, of the dimer.

$$\boldsymbol{H_0} = \sum_{i=1}^{N} E_0 \cdot \boldsymbol{E_{ii}}$$

Where $\boldsymbol{E_{ii}} = |i><i|$ is the projector on the eigenstate *i* of $\boldsymbol{H_0}$. $\boldsymbol{\mu}$ is the $N \times N$ matrix of the permanent $\{\mu_{ii}\}$, and transition $\{\mu_{ij}\}$, dipole moments.

$$\boldsymbol{\mu} = \sum_{i,j=0}^{N} \mu_{ij} \cdot \boldsymbol{E}_{ij}$$

with $\boldsymbol{E}_{ij} = |i><j|$. All the values of $\{\mu_{ii}\}$ and $\{\mu_{ij}\}$ were calculated as in ref. [5] except μ_{00}, the ground state permanent dipole moment, whose value was taken from

measurements inf ref.[4], $|\mu_{00}| \approx 26.8\ a.u.$ The Hamiltonian, $\boldsymbol{H}$ is diagonalized as a function of the static field strength $\mathcal{E}$ to yield the $\{E_i(\mathcal{E})\}$.

Figure S10 shows the energies $E_i(\mathcal{E})$ of the 24 FS states in the $\overline{D}$ =3.7 nm monomer ensemble for two limiting orientations relative to the applied static field: aligned (left) and anti-aligned (right). These configurations correspond to the maximum energy shifts induced by the bias. In Figure S10, the bright states are drawn with a full line, whereas the dark states are drawn with a dashed line. A dark state is defined as a state with $\mu_{0i} < 1\ a.u.$ Figure S11 provides a zoomed view of just the $1S_{3/2}$ band.

For a 10 V bias applied across a 300 nm gap, the resulting electric field is $3.3 \cdot 10^7\ V \cdot m^{-1}$, which produces a negligible shift of ~200 cm^{-1}. Within the estimated range of field $\mathcal{E}$ variation, the bias therefore does not lead to any significant modification of the FS energy levels.

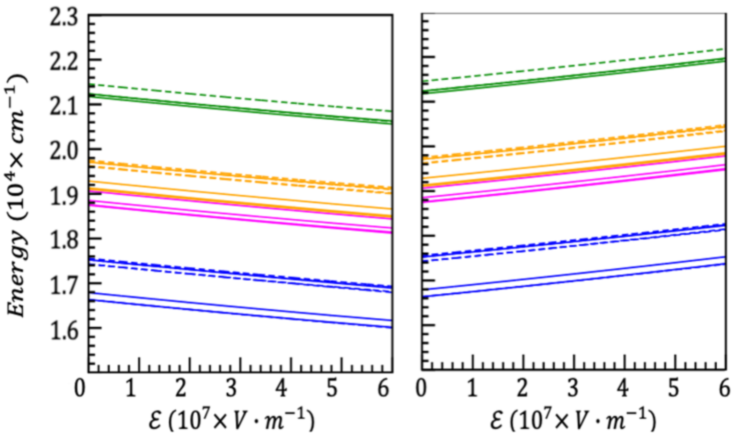

Figure S10. *$E_i(\mathcal{E})$ of the 24 FS monomer states ($\overline{D}$ =3.7 nm). The bands are denoted by different colors: $1S_{3/2}$ (blue); $1S_{1/2}$ (pink); $2S_{3/1}$ (yellow); $2S_{1/2}$ (green). The bright states are drawn with a full line, whereas the dark states are drawn with a dashed line. A dark state is defined as a state with $\mu_{0i} < 1\ a.u.$*

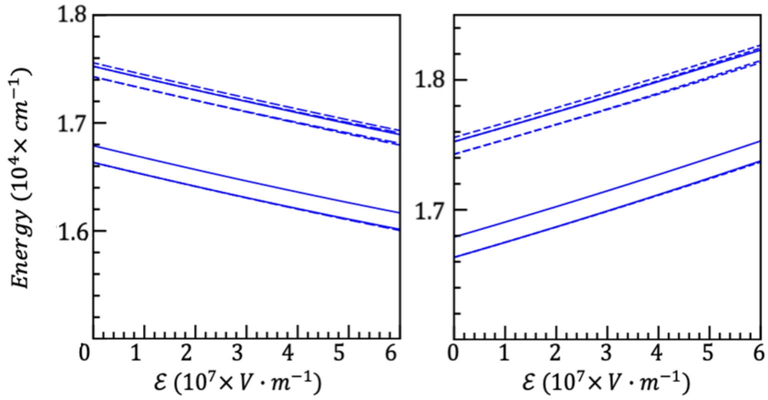

***Figure S11**. Zoom-in of the $1S_{3/2}$ band from Figure S9.*

S5. Electrostatic charging energy of CdSe quantum dots and comparison to the bias-induced energy scale

To evaluate whether the applied bias in our device can plausibly lead to charge injection and dot charging (a prerequisite for trion formation), we estimate (i) the single-charge addition (charging) energy of a CdSe QD and (ii) the electrostatic energy scale associated with the applied bias across the electrode gap.

Adding one excess electron (or hole) to an electrically isolated QD costs an electrostatic charging energy $E_c = e^2/4\pi\varepsilon_r\varepsilon_o D$.[6] Using $\varepsilon_o = 8.854\ 10^{-12}\ F/m$, $e = 1.602 \cdot 10^{-19} C$, a representative QD diameter $D = 3.7\ nm$, and $\varepsilon_r = 6$ (a representative value for a ligand-dominated environment,[7] we obtain $E_c \approx 65\ meV$.

We next estimate the electric field in the electrode gap under a typical applied bias. For a voltage drop V across a gap d, the electric field is $E = \frac{V}{d} \approx 3.3 \cdot 10^7\ \frac{V}{m}$ for $V = 10\ V$ and $d = 300\ nm$. The corresponding potential drop across a length scale comparable to one QD diameter D is $\Delta V_{QD} \approx E \times D \approx 1.22 \times 10^{-1} V$, which expressed as an energy scale for a single elementary charge become: $\Delta U_{QD} \approx e\Delta V_{QD} \approx 122\ meV$.

Comparing the two estimates, the bias-induced single-charge energy scale (~120 meV at 10 V across 300 nm) is larger than the single-charge QD charging energy (~70 meV). This supports the plausibility that, under applied bias, electrons can be injected and/or redistributed such that QDs can become transiently charged, enabling efficient exciton-to-trion conversion via interaction with excess carriers.

As a final note, it is important to stress that these estimates treat the QD as a spherical capacitor embedded in a uniform dielectric medium and assume that a substantial fraction of the applied voltage drops across the electrode gap. Local field enhancement, energetic disorder, and screening in the QD film can shift the effective values; nonetheless, the purpose here is just to establish the relevant order of magnitude.

Additional References

1. Klimov, V. I., McBranch, D. W., Leatherdale, C. A. & Bawendi, M. G. Electron and hole relaxation pathways in semiconductor quantum dots. *Phys. Rev. B* **60**, 13740–13749 (1999).

2. Collini, E. *et al.* Room-Temperature Inter-Dot Coherent Dynamics in Multilayer Quantum Dot Materials. *J. Phys. Chem. C* **124**, 16222–16231 (2020).

3. Hamilton, J. R. *et al.* Time–Frequency Signatures of Electronic Coherence of Colloidal CdSe Quantum Dot Dimer Assemblies Probed at Room Temperature by Two-Dimensional Electronic Spectroscopy. *Nanomaterials* **13**, 2096 (2023).

4. Collini, E., Gattuso, H., Levine, R. D. & Remacle, F. Ultrafast fs coherent excitonic dynamics in CdSe quantum dots assemblies addressed and probed by 2D electronic spectroscopy. *J. Chem. Phys.* **154**, 014301 (2021).

5. Gattuso, H., Fresch, B., Levine, R. D. & Remacle, F. Coherent Exciton Dynamics in Ensembles of Size-Dispersed CdSe Quantum Dot Dimers Probed via Ultrafast Spectroscopy: A Quantum Computational Study. *Appl. Sci.* **10**, 1328 (2020).

6. Cao, G. & Wang, Y. *Nanostructures and Nanotechnology*. (World Scientific, Singapore, 2011).

7. Markovich, G. *et al.* Architectonic Quantum Dot Solids. *Acc. Chem. Res.* **32**, 415–423 (1999).
